# Pachymeningeal carcinomatosis: an unusual location of metastization of adenoid cystic carcinoma

**DOI:** 10.1016/j.bjorl.2020.10.013

**Published:** 2020-11-23

**Authors:** André Pires, Cláudia Vieira, Manuel Jácome, Diana Moreira, Mavilde Arantes

**Affiliations:** a Instituto Português de Oncologia do Porto Francisco Gentil, Serviço de Radioterapia Externa, Porto, Portugal; b Instituto Português de Oncologia do Porto Francisco Gentil, Serviço de Oncologia Médica, Porto, Portugal; c Instituto Português de Oncologia do Porto Francisco Gentil, Serviço de Anatomia Patológica, Porto, Portugal; d Instituto Português de Oncologia do Porto Francisco Gentil, Serviço de Radiologia, Porto, Portugal

## Introduction

Adenoid cystic carcinoma (ACC) is a rare tumor, with a yearly incidence of 1.4 cases per million inhabitants in northern Portugal.^1^ It represents the most common malignant tumour of the submandibular glands (15%–30%).^2^ ACC most commonly affects the salivary glands (major and minor) but may arise in a wide variety of anatomical sites in and outside the head and neck, as the tracheobronchial tree, esophagus, breast, lungs, prostate, uterine cervix, and vulva.^3^ Histologically, there are three defined growth patterns used to describe this tumors: the cribriform, the tubular, and the solid pattern.^2^

ACC has been described as a tumor that grows and invades local tissues, with a high propensity for infiltrating through perineural invasion, but regional lymph node metastization is regarded as rare.^2^, ^3^ This way, free surgical margins are usually not achieved, despite the preoperative impression of experienced surgeons that complete resection is possible.^3^

This biological behavior translates into the characteristic development of late distant metastases (DM), despite the optimal treatment with combined surgery and radiotherapy (RT), with a favorable 5 year survival rate but poor long term survivals.^3^, ^4^ The role of chemotherapy (CTX) is still controversial but is generally recommended as a palliative treatment.^3^

The frequent development of distant metastases determines the unfavorable outcomes. Even when locoregional control has been achieved, patients frequently develop DM several years after the initial diagnosis.^4^ Several studies have attempted to determine the factors that influence it, with some inconsistent results. According to Shingaki et al.,^4^ the status of surgical margins appeared to be the only factor associated. The same authors reported that local recurrence did not affect the risk of distant metastization. The perineural invasion has also been implicated in predisposition for DM in some papers.^2^ DM occurs most frequently in the lungs, followed by bones, liver and, rarely, brain. The survival is significantly associated with the site of metastization and is better in patients with lung compared to bone and other organs.^2^

In this paper, we report a case of a patient with ACC that develops pachymeningeal carcinomatosis. Few cases of hematogenous DM of the dura by ACC have been published, however, as far as we are aware, this is the first one of a submandibular gland primary ACC.

## Case report

**Figure 1:**
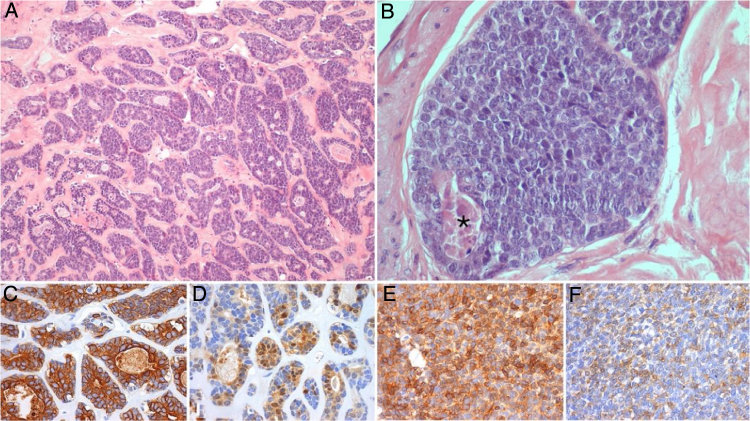
Representative photomicrographs of the primary submandibular tumor: (A and B) (Hematoxylin and eosin, ×100 and ×400) shows an high grade adenoid cystic carcinoma, with a cribriform and solid (this one representing more than 30% of the tumoral volume) patterns, with areas of necrosis (*); immunohistochemical stain for CK7 (C, ×200), PS100 (D, ×200), AML (E, ×200) and CD117 (F, ×200) demonstrate the biphasic cell population characteristic of this tumor, comprised of ductal and myoepithelial cells.

We report the case of a 56-year-old female patient who presented with a painless swelling on the left submandibular gland. After an aspiration biopsy compatible with a pleomorphic adenoma, she underwent a left submandibulectomy with lymph node dissection of the adjacent compartments. The definitive histological report showed a high-grade ACC, with a solid pattern present in more than 30% (Fig. 1); nodal metastasis was present.

**Figure 2:**
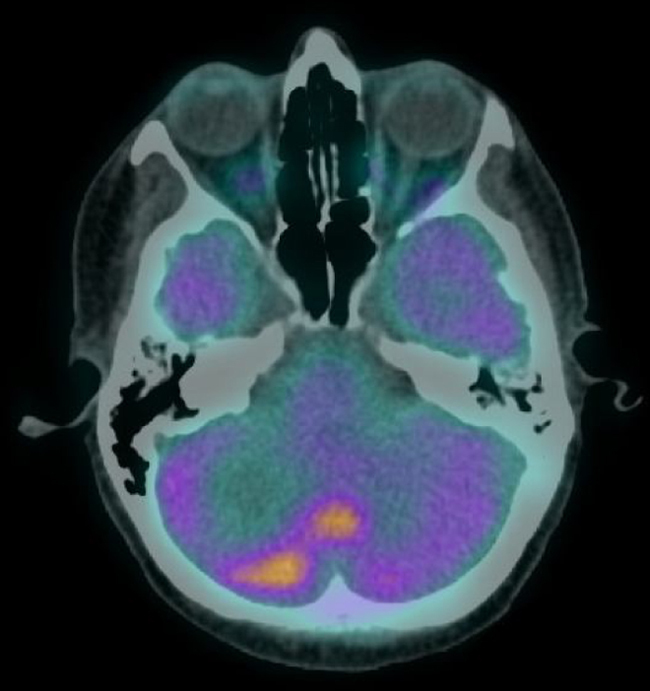
FDG-PET/CT showing FDG hypermetabolism in the right hemisphere and vermis of the cerebellum suggestive of secondary involvement.

**Figure 3:**
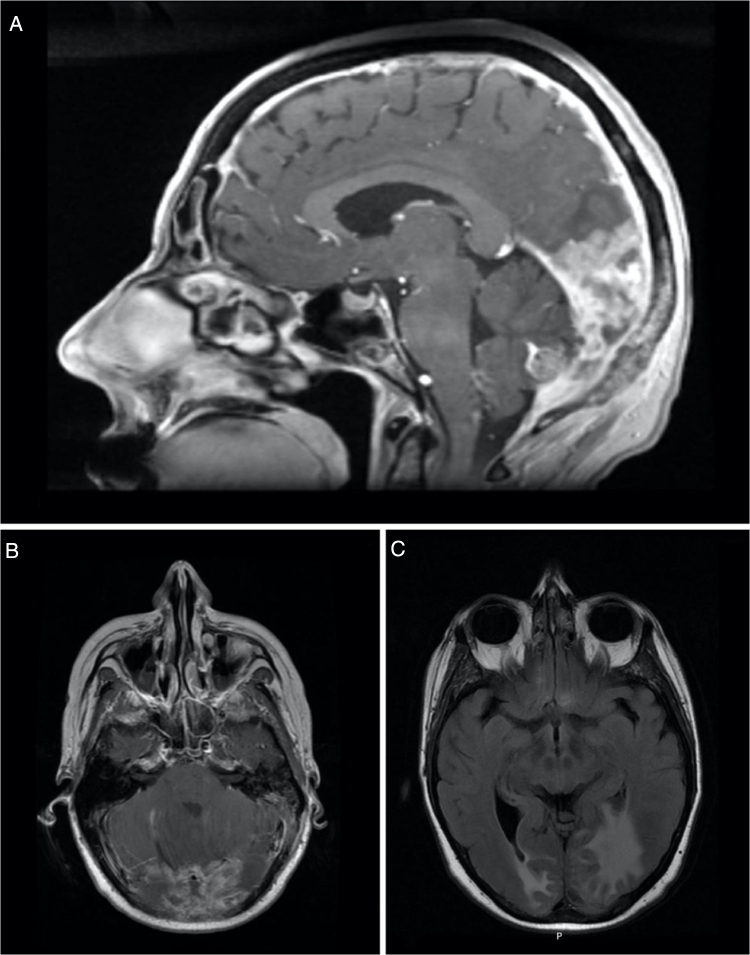
Sagital (A) and axial (B) contrast-enhanced T1-weighted MRI showing thickening and enhancement of the dura suggestive of pachymeningeal carcinomatosis (arrow). Axial (C) T2-FLAIR-weighted MRI showing vasogenic edema in the right occipital lobe and left occipito-temporal region.

The patient was staged as pT2N2a (ENE+) R1 (AJCC 8th edition) and was proposed for RT and radiosensitising CTX. She received 50–60 Gy to the base of the skull and left nodal levels IA to IV, and 66 Gy to the surgical bed (in 28 + 3 fractions, with VMAT-SIB technique), with concomitant cisplatin and was kept under surveillance. After 8 months, she was diagnosed with vertebral metastasis and a surgical procedure for vertebral stabilization of L3 and D8 was done. At that time, the FDG-PET/CT showed extensive bone involvement, so the patient was proposed for palliative RT to D8 and L3 (20 Gy in 5 fractions, 3DRT technique) and started CTX with carboplatin and paclitaxel, and bisphosphonates. After completing the 6 cycles, the FDG-PET/CT revealed worsening of the bone metastasis and liver metastasis, and the patient started a 2nd line of CTX with gemcitabine. Three months later, the FDG-PET/CT questioned the existence of metastatic cerebellar involvement (Fig. 2), and the MRI showed pachymeningeal carcinomatosis of the posterior fossa, right occipital and left occipito-temporal regions, with involvement of the adjacent bone and cerebellum (Fig. 3).

The neurological examination showed only a slight ataxia and hypoesthesia of the left thigh. The patient was proposed for whole brain RT and received 30 Gy in 10 fractions, and then started CTX with pegylated liposomal doxorubicin. In the following months, the patient remained under CTX and presented neurologically stable, with no evidence of clinical intracerebral condition worsening. The patient died of multiple systematic metastasis 4 months after finishing RT.

## Discussion

The overall frequency of intracranial invasion of ACC has been reported to be between 4% and 22%.^5^ A review of the world literature has revealed a wide variety of intracranial metastization of ACC. Overall, this involvement can occur in one of three ways: direct extension, perineural spread and hematogenous spread.^6^

Direct extension and perineural spread have long been recognized as methods of spread to locations of the intracranial metastatic lesions. This explains why most intracranial metastasis are located at the base of the skull, having extended transcranially directly or perineurally along cranial nerves from the primary lesions in neighbor structures as nasopharynx, paranasal sinus, and the lacrimal gland. Even though hematogenous metastasis to liver, lung and bone are reasonably common in the late course of this disease, true hematogenous metastases to the intracranial compartment are rare.^5^, ^6^

We present a patient with a presumed hematogenous extensive metastases to the dura from a submandibular gland ACC. In this case, the hematogenous route must be invoked to explain the location of the lesion. The lesion in our case, while partially extra-axial, is in an anatomic position inconsistent with retrograde perineural spread from the primary site in the submandibular gland and, furthermore, MRI showed no evidence of contiguous spread of the tumor to support direct extension across the base of the skull.

The few reports existent in the literature consistent with hematogenous intracranial spread are, in the majority, in a form of parenchymatous metastasis, from primaries tumors from the breast,^7^ the parotid,^8^, ^9^ the lung,^10^ the skin,^11^ the Bartholin’s gland,^12^, ^13^ and unknown primaries.^14^ As far as metastization to the dura, there is only one published case, from the parotid gland.^15^

## Conclusion

Hematogenous intracranial metastization is a particularly unusual way of spread of this tumor, with a narrow variety of primary sites and intracranial locations reported. No cases of involvement of the dura from a submandibular gland primary are reported in the literature, making this the first report of dura involvement of ACC from the submandibular gland.

## Conflicts of interest

The authors declare no conflicts of interest.
